# A Simulation-Based Approach to Procedural Education in Resource-Limited Settings: Designing an Abdominal Trainer for Paracentesis and Lumbar Puncture

**DOI:** 10.7759/cureus.101676

**Published:** 2026-01-16

**Authors:** Alexander Hayden, Sifat M Alam, Elena M Willow, Brian F Quach, Andrew Eyre

**Affiliations:** 1 School of Medicine, Frank H. Netter MD School of Medicine, Quinnipiac University, North Haven, USA; 2 Surgery, Frank H. Netter MD School of Medicine, Quinnipiac University, North Haven, USA; 3 Medicine, Frank H. Netter MD School of Medicine, Quinnipiac University, North Haven, USA; 4 Emergency Medicine, Brigham & Women’s Hospital, Harvard Medical School, Boston, USA

**Keywords:** abdominal paracentesis, educational equity and access, emergency medicine, general internal medicine, global health education, low- and middle-income countries (lmic), lumbar puncture (lp), medical simulation, procedural education, resource-limited setting

## Abstract

In recent years, advancements in medical simulation training and technology have significantly enhanced medical education and practice in high-income countries (HICs), enabling clinicians to approach performing complex and invasive procedures with greater confidence and skill. Improved procedural training through simulation may contribute to reduced mortality rates and improved clinical outcomes in HICs.

Two common truncal procedures include paracentesis for the management of abdominal ascites and lumbar puncture (LP) for the diagnosis of infectious etiologies. Paracentesis and LP remain common procedures across multiple specialties; however, high-quality training opportunities often entail significant costs. Challenges such as financial constraints and supply chain disruption may limit access to simulators for clinicians practicing in low- and middle-income countries (LMICs).

To address this gap in training opportunities, we designed a customizable, cost-effective, and reusable simulator that enables the practice of abdominal procedures, such as paracentesis and LP, for clinicians working in remote areas and LMICs. This trainer may allow providers in LMICs and under-resourced areas to improve procedural training and ultimately provide better care to their patients. The design and implementation of our simulator may improve access to procedural education opportunities and, ultimately, contribute to improved procedural confidence, speed, accuracy, and comfort among clinicians working in these areas.

## Introduction

Low- and middle-income countries (LMICs) are endemic to many infectious diseases, which may require invasive procedures for diagnosis and treatment, such as paracentesis for spontaneous bacterial peritonitis, secondary to accumulating ascites, or lumbar puncture (LP) for cerebrospinal fluid analysis (CSF) [1-3]. Paracentesis is a fundamental procedure needed for the management and continued care of patients with abdominal ascites. Utilizing this technique, clinicians may evaluate the ascitic fluid, utilize the serum-ascitic albumin gradient, and incorporate guideline-directed clinical care [4]. Additionally, LP is a procedure that has significance in the diagnosis, treatment, and continued management of a large variety of neurologic disorders. It is important for the acquisition of CSF, medication administration, and intracerebral pressure measurement [5]. When performed correctly by a well-trained clinician, the procedure is generally well tolerated and has a low rate of complications [6]. Care must be taken when performing these procedures, as the abdominal and spinal regions contain delicate structures that may result in serious complications if they are performed incorrectly.

Simulation-based training has been shown to improve procedural performance and operative preparedness among surgeons and physicians in resource-limited countries [7-10]. Procedural simulation specifically provides clinicians with the opportunity to develop and refine practical skills prior to their application in real patient care. A common challenge limiting the use of procedural simulations in LMICs is financial constraint and supply chain disruption. Equipment cost, access to contemporary training resources, and clinical expertise are major limiting factors in providing effective simulation training to students and clinicians in LMICs [11-13].

These findings highlight the need for affordable, hands-on simulation technology in resource-limited settings to expand training capacity and improve access to care. In this article, we detail the methodology of designing an abdominal simulator capable of teaching paracentesis and LP to learners in resource-limited environments.

## Technical report

**Figure 1:**
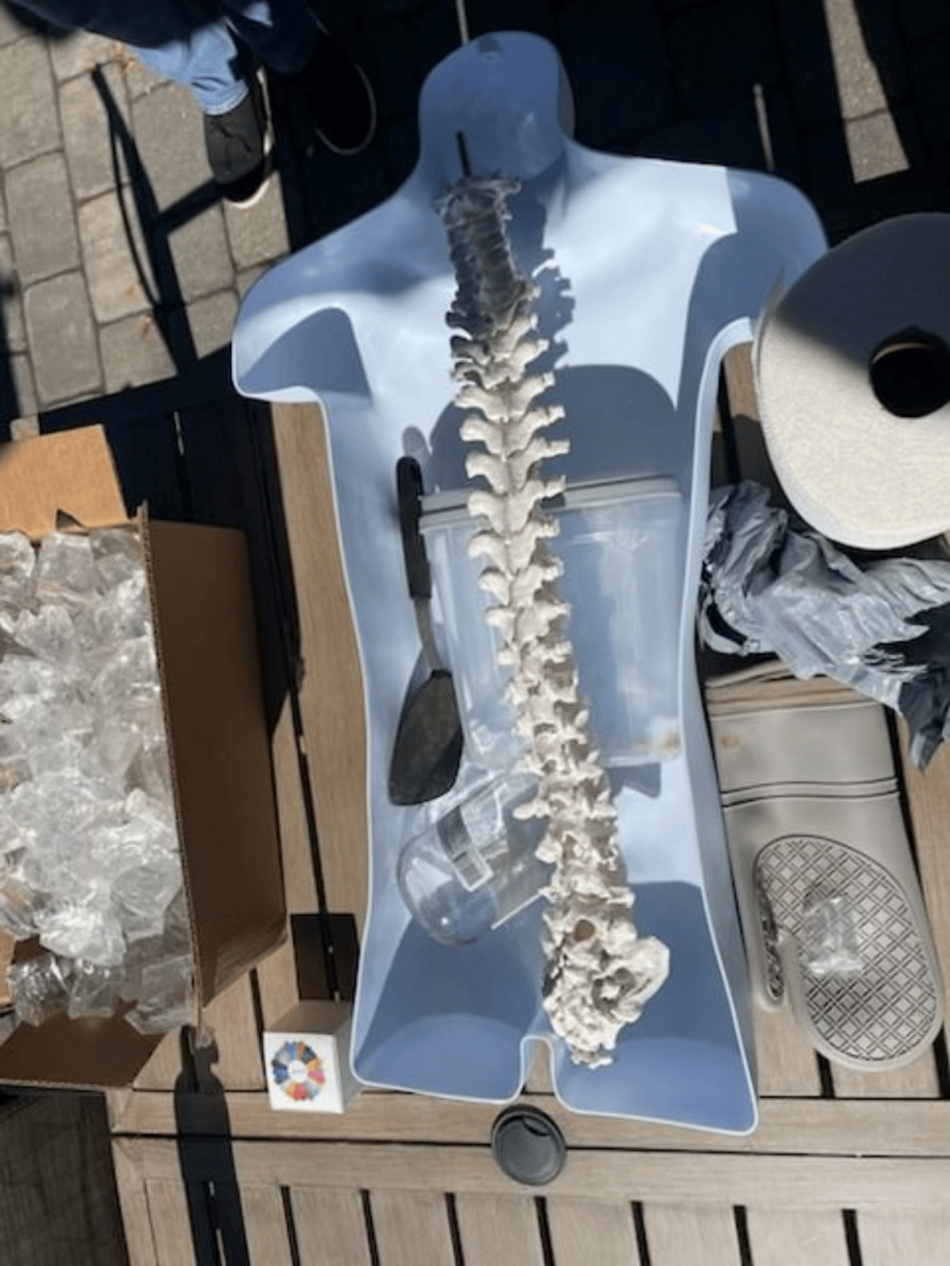
Component Materials and Mold for Simulator

The objective of this project was to design and construct a customizable, cost-effective, and reusable simulator capable of simulating both paracentesis and LP procedures, which enables the practice of abdominal surgical procedures for clinicians working in remote areas and LMICs. The simulator was created using low-cost, readily available materials (Table 1). It was constructed using a clothing manikin torso and a lumbar spine model as a base (Figure 1). Ballistic gel was melted in a low-cost stock pot on an outdoor grill until a consistency suitable for molding within the abdominal mannequin was reached. Alternative methods of liquifying the gel may include electric slow cookers or heating over fire pits. Beige dye was incorporated into the gel to approximate a Caucasian skin tone for opacity.

**Table 1:** Total Cost of Materials Note: Items are typically bought in bulk, and individual units are used to create the model. Over time, the costs of components are subject to change. The total cost of the design may vary depending on the manufacturer, retail price, and quantity of items. The specific brands of components listed above are not essential for creation but simply reflect what we used to design the simulator. Prices are reflective of USD as of December 2025.

Model Components	Cost per Unit (in USD)	Amazon Standard Identification Number (ASIN)
Ballistic Gel Block (x2)	$232.00	B0B9YNSFYP
Flesh Colored Dye	$9.00	B0D81HQ8NB
Abdominal Mold Mannequin	$37.00	B0842YBWK8
3 mm Artificial Skin (x1)	$1.90	B0CZ6ZNBJ7
Total Cost of Design	$279.90	

**Figure 2:**
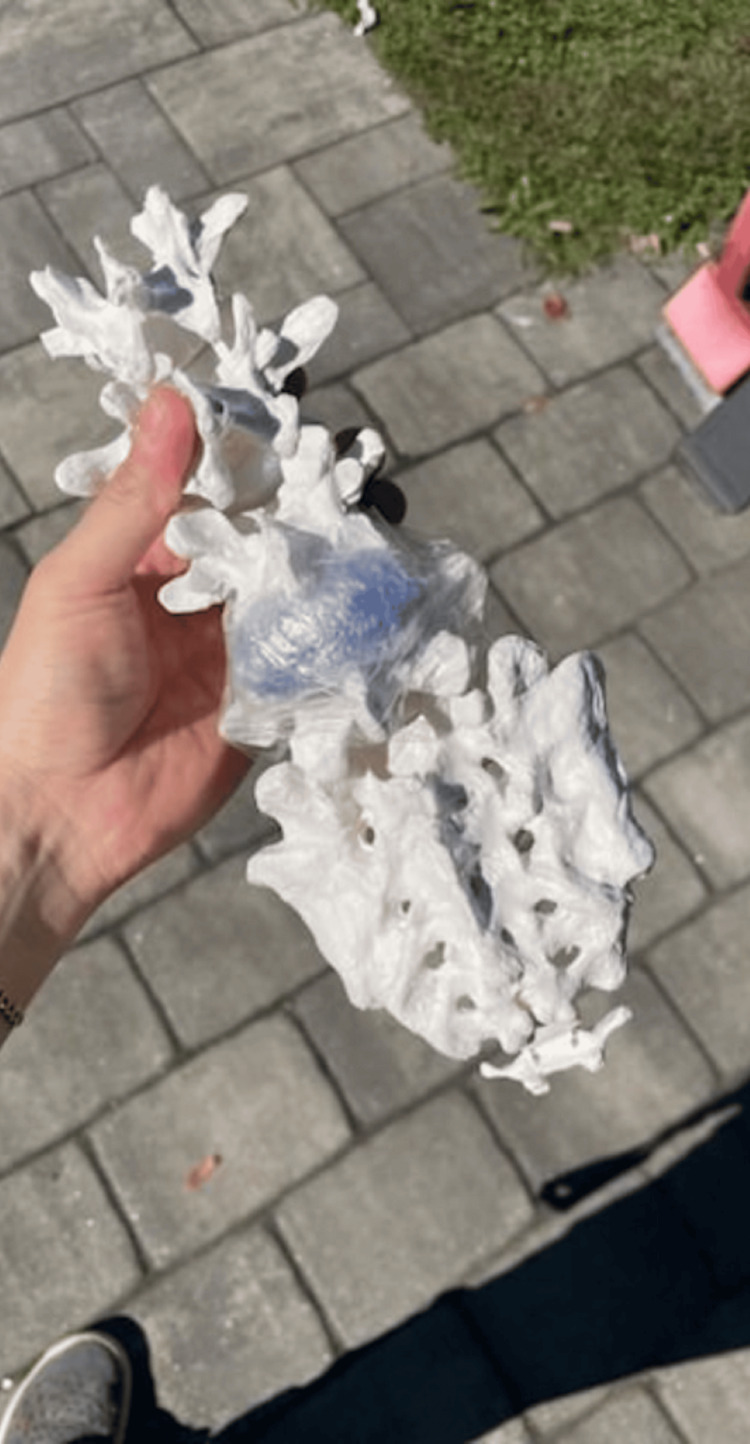
Spine With Lumbar Puncture Fluid Reservoir

**Figure 3:**
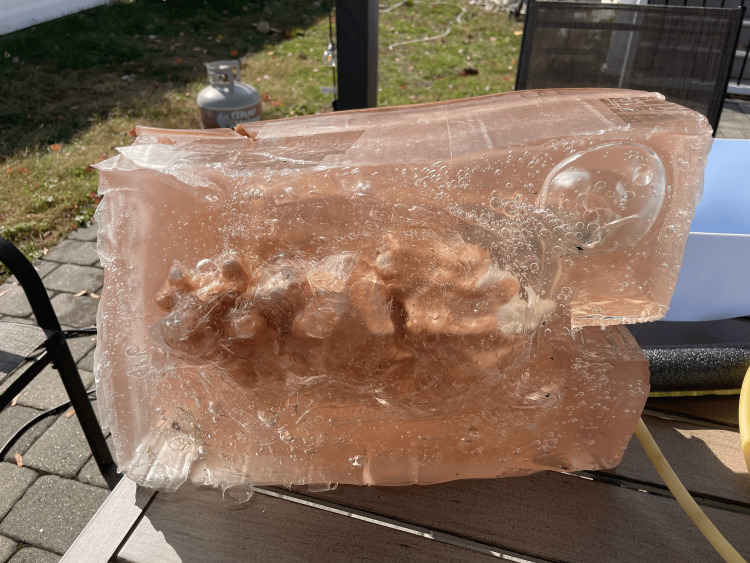
Posterior Aspect of Simulator Before procedural practice, tattoo skin should be applied to enhance fidelity and opacity.

A plastic food container was positioned inside the mannequin to serve as a placeholder for the abdominal cavity. The gel was then poured into the mannequin to cover the container. While the gel cooled, a partial spine replica, repurposed from a previous project, was modified to simulate a fluid-filled spinal column suitable for LP training [14]. This was achieved by inserting a balloon filled with water, securely tied with plastic wrap and taped into the L4 position on the model (Figure 2). For the purposes of this project, the balloon was constructed from repurposed nitrile gloves. The glove fingers were cut, filled with water, and tied off from the top to conserve resources. The spine was then placed in the abdominal mannequin model at the anatomically correct location, and additional gel was poured to cover it (Figure 3). After approximately four hours of cooling, we removed the abdominal gel from the mold, resulting in a formed abdomen with the spine embedded. A reusable synthetic skin layer was placed over the spine to simulate human skin for the LP procedures. 

**Figure 4:**
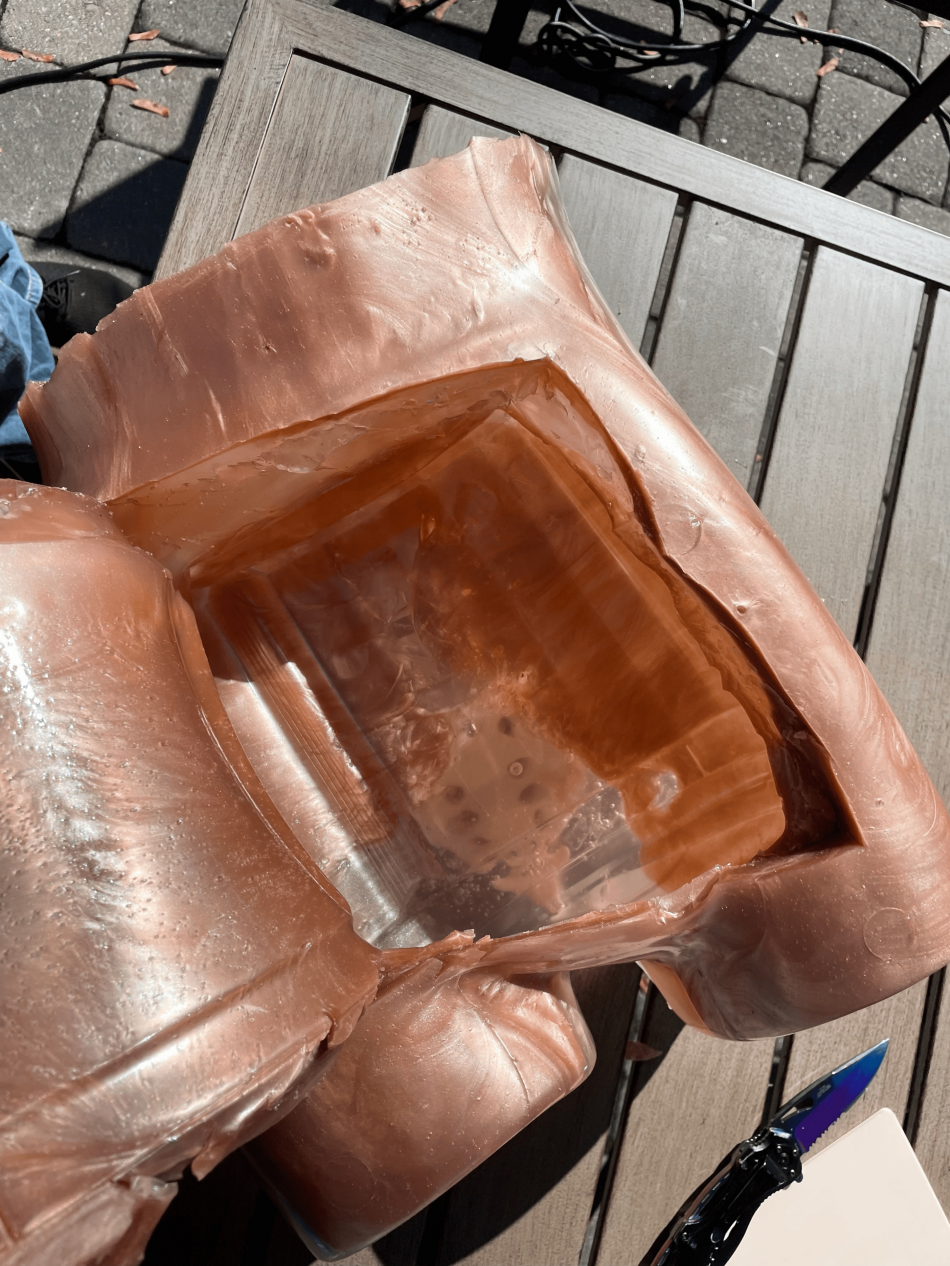
Abdominal Cavity

**Figure 5:**
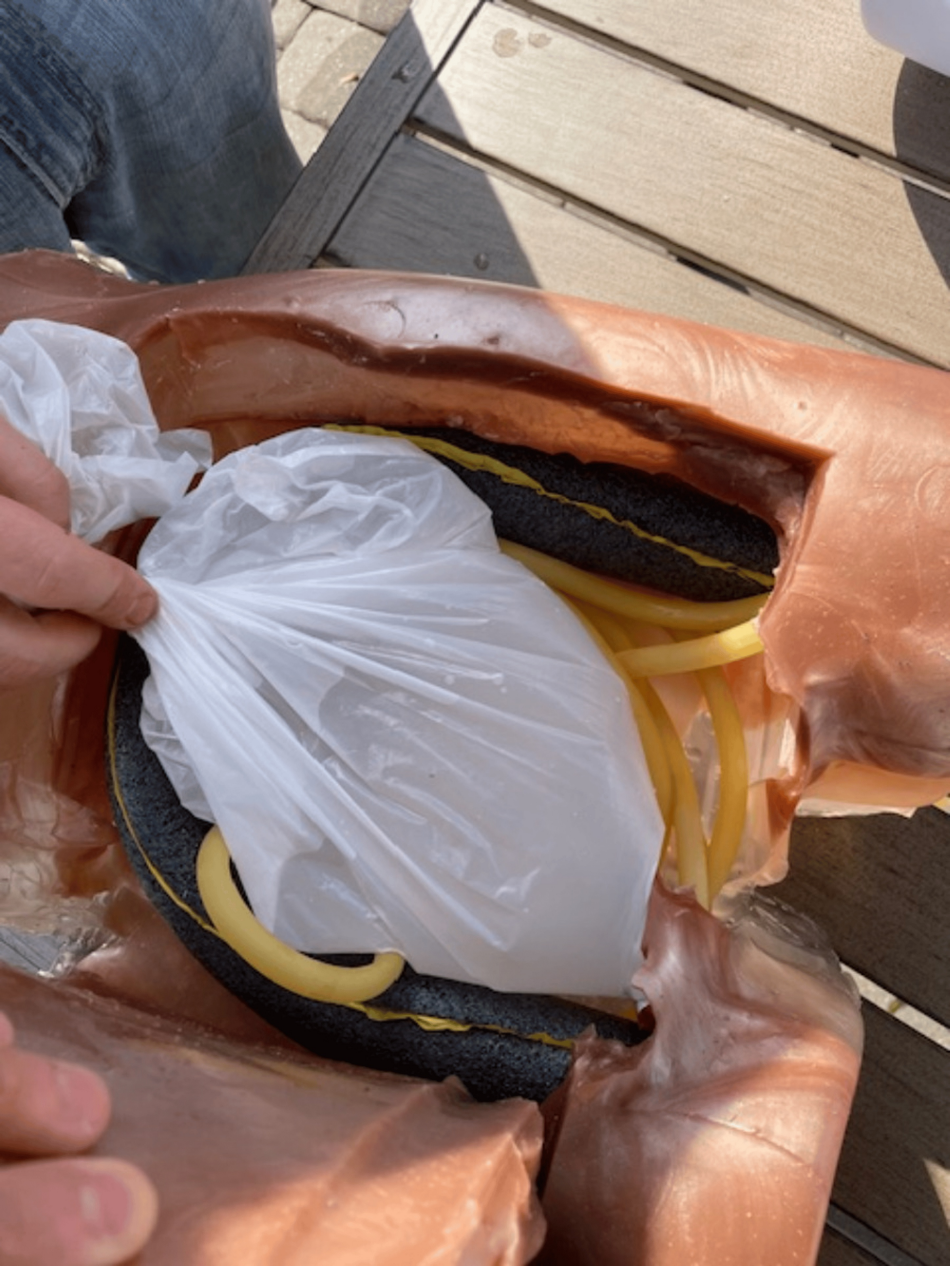
Abdominal Cavity With Simulated Intestines and Fluid Reservoir The simulated bowel, shown in this figure, was included for illustrative purposes and is not part of the final product.

A U-shaped incision was then made on the anterior portion of the abdomen to create a flap, allowing for the extraction of the plastic food container, which was subsequently removed, leaving a well-defined abdominal cavity (Figure 4). Within the abdominal cavity, a garbage bag filled with 500 mL of water was added to replicate ascites for paracentesis training (Figure 5). Additional gel was melted using the same method as before and poured back over the incision to recreate the abdominal flap for the extraction of the food container and the placement of intestines and the bag of water.

Results

**Figure 6:**
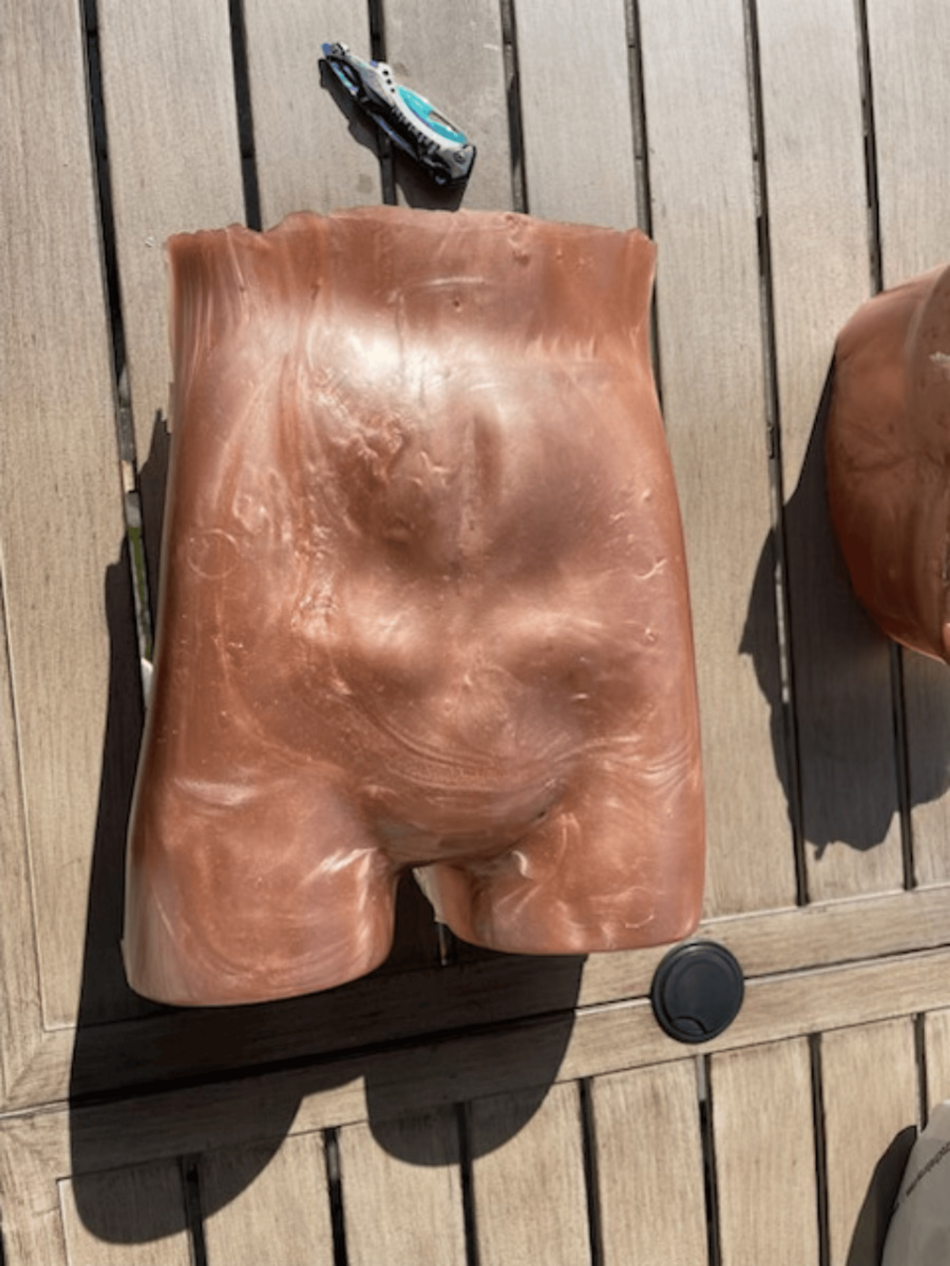
Anterior Aspect of Simulator

**Figure 7:**
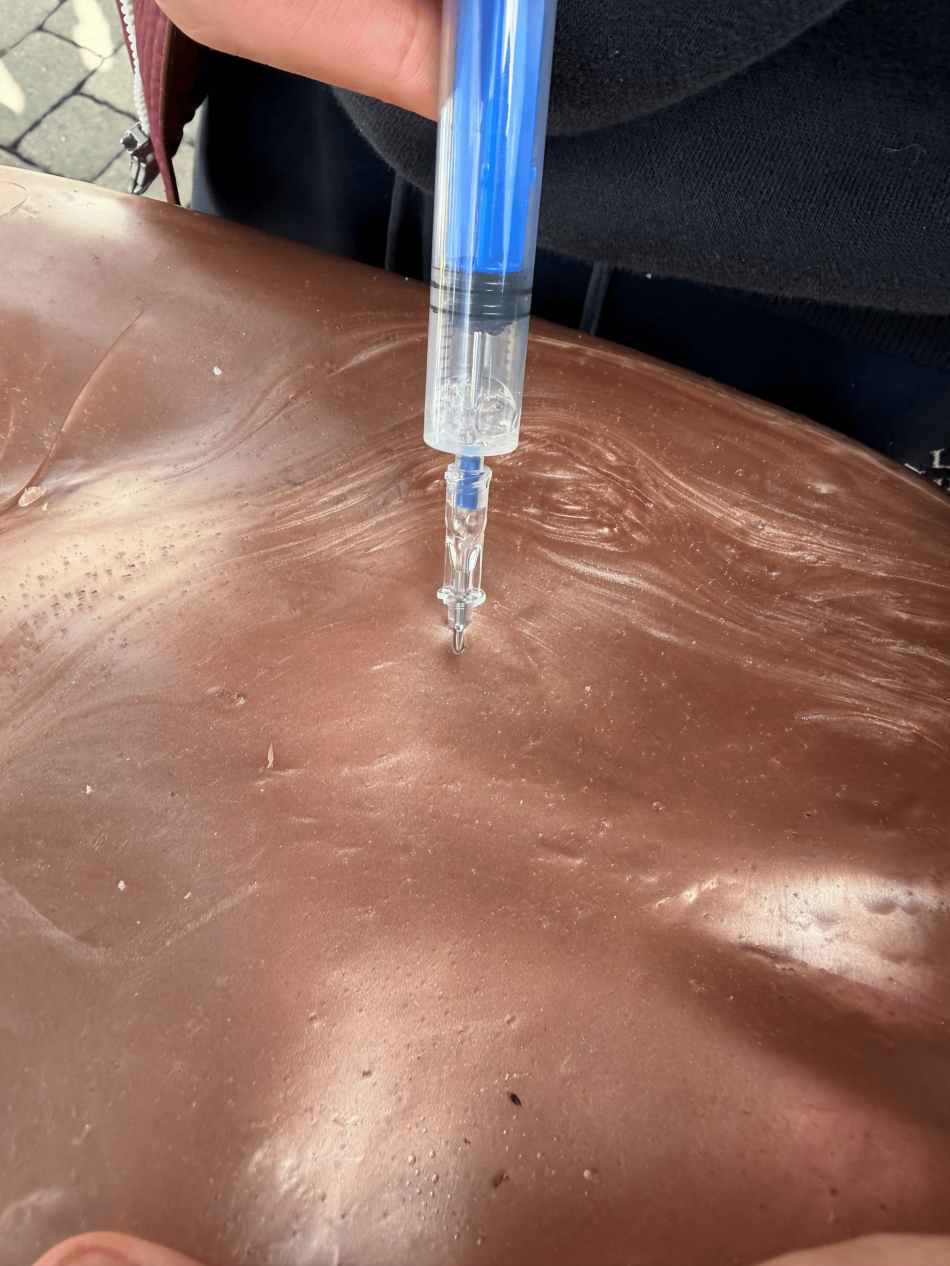
Paracentesis Function

**Figure 8:**
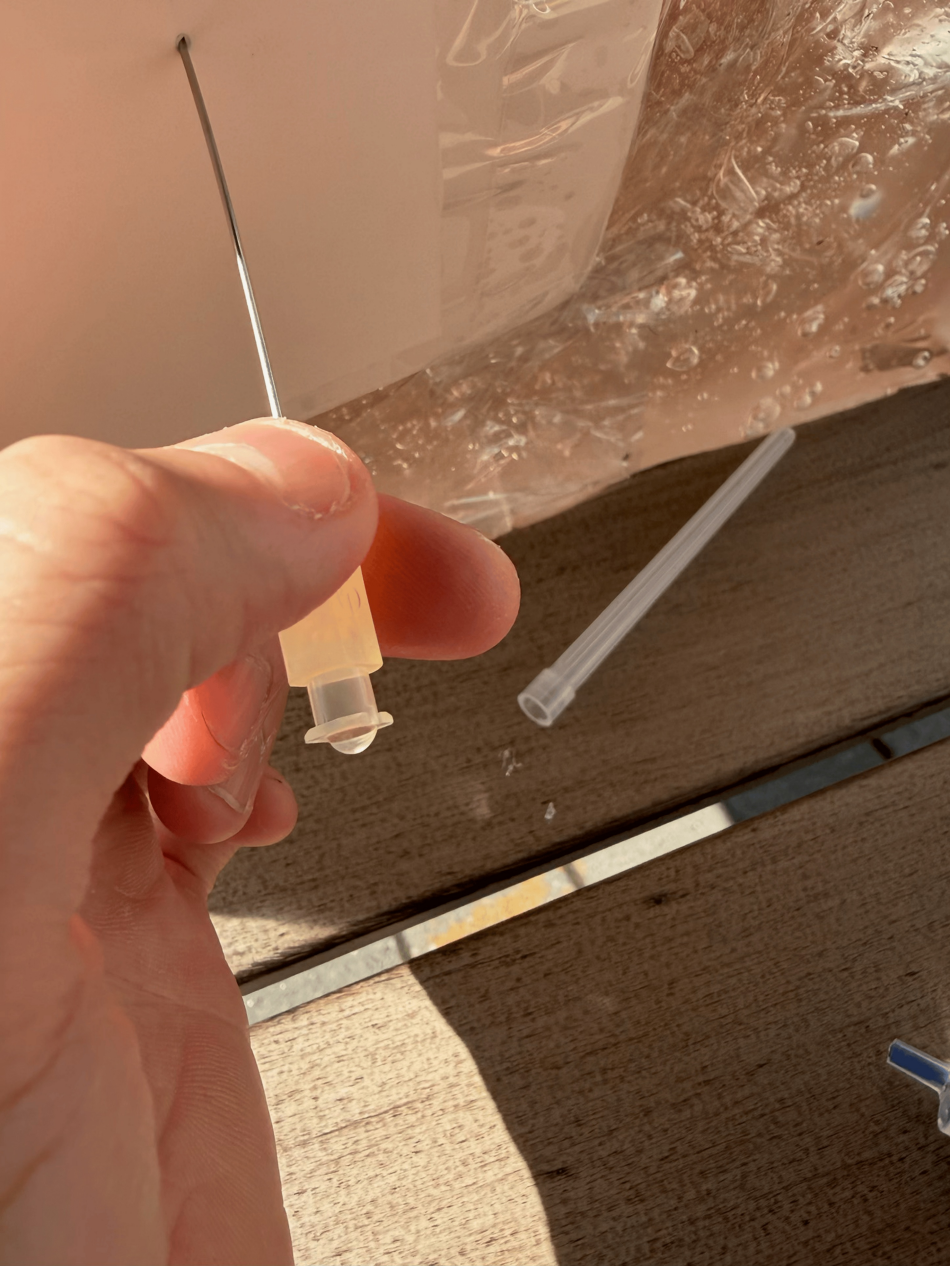
Lumbar Puncture Function

The completed simulator was then utilized to practice both paracentesis and LP. Paracentesis was performed using a standard technique, allowing learners to realistically aspirate ascitic fluid. When placed in the lateral decubitus position, the model was used to replicate an LP. Utilizing standard equipment and technique, learners were able to obtain simulated CSF at the L4-L5 space. Using repurposed, cost-effective materials, we created a simulator that replicates a human abdomen for approximately $279.90 USD (Figure 6). This model allows trainees to practice the procedural skills that pertain to the abdominal region, particularly paracentesis and LP. Paracentesis may be performed using a landmark-guided approach; when the trainee successfully punctures the fluid reservoir, they are able to aspirate fluid (Figure 7). The lumbar spine may be palpated through the opaque ballistic gel and the tattoo skin layer, which is adhered over the top to prevent visualization of the spine. Underneath the fourth vertebra of the lumbar spine, the balloon may be accessed for fluid return (Figure 8).

## Discussion

Our model for this procedural simulator, built from cost-effective and repurposed materials, carried a net cost of $279.90 USD. We were able to develop a sustainable, reusable simulation model capable of supporting both paracentesis and LP training. Although our lumbar spine was repurposed from a prior project, life-size commercial products may cost approximately $30 USD on Amazon at the time of article publication. Life-size spinal column models, as an alternative, may incur higher costs but may also be beneficial for institutions planning to construct additional simulation models utilizing these parts. Our largest cost contributor to this model was the ballistic gel. Construction required two 7.73 kg blocks to properly fill our mold for the entire abdominal cavity. Ballistic gel serves as the primary tissue analog due to its favorable tactile properties, durability, as well as excellent ability to be rapidly melted down and reconstructed in any shape we require. It also has exceptional ultrasound compatibility, which may be adapted if this were an important learning object in the future for practicing point-of-care ultrasound (POCUS) or focused assessments with sonography for trauma (FAST) examinations.

Development of this dual-purpose model was driven by the need to provide affordable, functional, and reusable trainer models for these procedures. Although high-fidelity commercial trainers exist, some models may cost upwards of $6,000 USD, not including replacement parts. Additionally, these trainers are typically limited to a single procedure, further constraining access to adequate training opportunities. Our model provides affordability, while also offering functionally realistic alternatives that preserve educational value and substantially lower financial barriers in comparison to commercial trainers. Given the material’s physical properties, the ballistic gelatin may be further repurposed for the development of simulation models for different procedures [14]. When considering the reusability of our model, the design enables users to replace fluid reservoirs by extracting the emptied reservoir from its respective location on the model. This process requires reheating ballistic gel and pouring it over the incisions to create a seal for needle puncture. This maintenance procedure may take 20-30 minutes per cycle. 

This model will allow learners across multiple skill levels to practice and hone their skills in LP and paracentesis, which are invasive procedures that are commonly performed. Each can aid in the diagnosis and therapeutic relief of multiple pathological conditions for patients living in LMICs. The simulator supports the landmark approach as well as ultrasound-guided techniques for paracentesis, which can assist learners in reinforcing proper probe positioning, needle visualization, and depth control [15]. These are skills that must be learned by future practitioners to avoid vascular, bowel, or other solid organ injuries [16]. 

LP is also a capability of our simulator. The combination of the synthetic skin over the top of the artificial spine allows the user to properly palpate landmarks and acquire the correct location for the procedure to be performed. Synthetic skin and ballistic gel provide realistic resistance during needle advancement, adding fidelity to our simulator. The combination of aluminum foil and plastic wrap reliably simulates the characteristic “pop” sensation experienced by practitioners during LP when penetrating the ligamentum flavum, depending on the number of layers applied to the balloon.

Limitations

Limitations of this model may include the inability to practice all possible thoracoabdominal or surgical procedures, as the model currently stands. As designed, it is specifically intended for the practice of LP and paracentesis. Expanding the model’s capabilities to include common techniques imperative for general surgeons, such as laparoscopic and gross exploration, thoracoabdominal hemorrhage control, and appendectomy, shows promise. This may be accomplished with the introduction of additional organ structures, such as the aorta, liver, and appendix, within the cavity. Although this model was developed from the perspective of physicians performing invasive procedures, there remains a substantial opportunity to expand the simulator’s capabilities for use in surgical training in LMICs. 

Secondly, given the tactile properties of the ballistic gel, it is particularly challenging to create abdominal distention that is characteristically seen in a patient who presents with typical ascites. The simulator maintained a nondistended shape even when the cavity was filled entirely with water, without the use of a garbage bag adjunct. For hygiene and cleanliness, we opted to use a garbage bag as the fluid reservoir in the cavity, despite the nondistention and slight leakage from the initial puncture. This adjunct preserved the accuracy of the fluid drainage target within the simulator in both supine and lateral decubitus positions. Future designs may explore the use of alternative materials, such as silicone, to achieve a high-fidelity, distended abdominal appearance. Additionally, future designs may explore alternative options for developing an LP adjunct with a larger fluid reservoir, rather than using a glove tip filled with water. In future studies, we aim to invite a large, diverse group of learners who regularly perform these procedures to ascertain the benefits of practicing on our model.

## Conclusions

In summary, utilizing readily accessible and repurposed materials, we created an abdominal simulator for approximately $279.90 USD for paracentesis and LP procedural practice. The model may be a viable, cost-effective alternative to commercial products. Further evaluation and subject matter expertise are vital in determining this model’s effectiveness as a training adjunct in LMICs. In future studies, we plan to invite clinicians who routinely perform LP and paracentesis to evaluate the model and provide structured feedback.
